# Assessment of mRNA Vaccine Immunogenicity in Solid Organ Transplant Recipients

**DOI:** 10.3390/medicina59061075

**Published:** 2023-06-02

**Authors:** Paraskevi Tsoutsoura, Efstathios Xagas, Sotirios Roussos, Angelos Hatzakis, Polyxeni Gourzi, Ioannis N. Boletis, Smaragdi Marinaki

**Affiliations:** 1 Clinic of Nephrology and Renal Transplantation, Laiko General Hospital, Medical School of Athens, National and Kapodistrian University, 11527 Athens, Greece; 2 Department of Hygiene, Epidemiology & Medical Statistics, School of Medicine, National and Kapodistrian University of Athens, 15772 Athens, Greece; 3 Molecular Immunopathology and Histocompatibility Unit, Onassis Cardiac Surgery Center, 17674 Athens, Greece

**Keywords:** mRNA vaccines, solid organ transplant recipients, SARS-CoV-2

## Abstract

*Background and Objectives:* Solid organ transplant (SOT) recipients have a higher risk of suffering from severe Coronavirus (COVID-19) compared to the general population. Studies have shown impaired immunogenicity of mRNA vaccines in this high-risk population; thus, SOT recipients have been prioritized globally for primary and booster doses. *Materials and Methods:* We analyzed 144 SOT recipients who had previously received two doses of BNT162b2 or mRNA1273 vaccine, and who were subsequently vaccinated with a booster dose of the mRNA1273 vaccine. Humoral and cellular immune responses were measured 1 and 3 months after the second dose, and 1 month after the third dose. *Results:* One month after the second dose, 33.6% (45/134) of patients displayed a positive antibody response with a median (25th, 75th) antibody titer of 9 (7, 161) AU/mL. Three months after the second dose, 41.8% (56/134) tested positive with a median (25th, 75th) antibody titer of 18 (7, 251) AU/mL. After the booster dose, the seropositivity rate increased to 69.4% (93/134), with a median (25th, 75th) titer of 966 (10, 8027) AU/mL. The specific SARS-CoV-2 T-cell response was assessed in 44 randomly selected recipients 3 months after the second dose, and 11.4% (5/44) of them had a positive response. Following the third dose, 42% (21/50) tested positive. Side effects after the third dose were mild, with pain at the injection site being the most frequent adverse effect, reported by 73.4% of the recipients. *Conclusion:* Our study shows a mild delayed increase in antibody titer, three months after primary vaccination compared to one month after. It also shows a robust augmentation of humoral and specific T-cell responses after the booster dose, as well as the safety and tolerability of the mRNA vaccines in SOT recipients.

## 1. Introduction

The coronavirus pandemic of 2019 (COVID-19) has caused a worldwide health crisis, with a tremendous effect on patients and healthcare systems. As of March 2023, the World Health Organization (WHO) reported over 761 million confirmed cases of COVID-19 worldwide and over 6.8 million deaths.

The implementation of vaccinations against SARS-CoV-2 has been an important milestone in the struggle against the pandemic, with over 13 billion vaccine doses administered globally [1]. Solid organ transplant (SOT) recipients are at an increased risk of severe disease and death from COVID-19, and thus were prioritized for vaccination early during the pandemic [2,3]. However, early studies have shown that the antibody response to COVID-19 vaccines in transplant recipients is impaired compared with the general population [4]. These findings were not unexpected, as older studies have suggested that immunocompromised patients, such as SOT recipients, develop impaired immunogenicity to vaccination against viral pathogens and low overall response rates to other vaccines [5,6]. SOT recipients have also demonstrated a similarly decreased T-cell immune response in both overall and case-control studies [7]. Recent published results reveal that kidney transplant recipients presented suboptimal responses after any vaccination schedule (initial, third and fourth dose) [8].

However, available evidence suggests that providing a booster dose of mRNA vaccine to SOT recipients who have previously received two doses of mRNA vaccine can increase antibody titers against SARS-CoV-2 [9,10]. In terms of heterologous vaccination, combining mRNA and viral vector vaccines has also been assessed, but not with better results than the homologous mRNA-based strategy [11].

This study aimed to investigate our main hypothesis that humoral and specific cellular responses enhance after a booster dose of mRNA vaccine in SOT recipients. Furthermore, we present data regarding the safety and adverse effects of vaccination with the booster dose, as well as results regarding response one and three months after primary doses administration.

## 2. Materials and Methods

### 2.1. Patients

Of our initial cohort of 455 solid transplant recipients, not all achieved success in following the study’s strict time frame for booster vaccination and blood sampling. Consequently, we included 144 SOT recipients who had previously received two doses of either BNT162b2 (Pfizer–BioNTech) or mRNA1273 (Moderna) vaccine, and were subsequently vaccinated with a booster dose of the mRNA1273 vaccine and who also attended all blood sample measurements [12]. We recruited patients from two referral hospitals (Laiko General Hospital of Athens and Onassis Cardiac Surgery Center) and collected data on demographics, vaccination-related side effects after each dose, and previous COVID-19 from questionnaires. The enrollment of patients started after the completion of primary vaccination. Participants were scheduled for 3 consecutive blood specimens to be collected 1 and 3 months after the second vaccine dose, as well as 1 month after the third dose, to determine the humoral response. SARS-CoV-2 specific T-cell response was also evaluated in a randomly selected subgroup, due to the complex and demanding procedure needed for this evaluation. Previous COVID-19 was not an exclusion criterion for blood sampling, because patients were advised to proceed with a full vaccination schedule two months after recovery. We obtained clinical data on transplantation status, immunosuppression, comorbidities, and concomitant medications from the medical charts. The estimated glomerular filtration rate (eGFR) was calculated using the Chronic Kidney Disease Epidemiology Collaboration (CKD-EPI) equation.

### 2.2. Blood Sampling

To assess the immunity response, we used blood samples taken at a median (25th, 75th) of 29 (22, 23.5) days and again at 85 (82, 91) days after the second dose. We also took blood samples following administration of the third dose at a median (25th, 75th) of 187 (182, 189) days after the second dose and 33 (30, 35) days after the third dose. For humoral assays, 5 mL blood was collected from each participant. The samples were centrifuged to separate the plasma. From participants where cellular assays were performed, 5 mL more blood was collected.

### 2.3. Humoral Response

Antibodies were measured using a chemiluminescent microparticle immunoassay (CMIA), which quantifies IgG antibodies against the receptor-binding domain (RBD) of the S1 subunit of the spike protein (Abbott SARS-CoV-2 IgG II Quant). The linear range of the assay was between 21 and 40,000 arbitrary units per milliliter (AU/mL) and the lower limit of detection (LoD) was 6.8 AU/mL [13]. Anti-SARS-CoV-2 RBD IgG assays showed an excellent correlation with neutralizing antibodies in early studies, which, however, used psedo-virus [14]. The evidence supports this correlation not only against the early variant but also against later variants such as Delta [14,15].

We also measured IgG antibodies against the SARS-CoV-2 nucleocapsid (N) protein, indicative of previous infection [16]. Patients who tested positive for anti-N antibodies were excluded from further analyses.

### 2.4. Cellular Response

We measured SARS-CoV-2-specific T-Cell responses only in a randomly selected subset of the study participants. Cellular immunity was assessed using the Quantiferon SARS-CoV-2 interferon-γ release assay (Qiagen), which detects interferon-γ (IFN-γ) secretion by CD4+ and CD8+ lymphocytes in response to SARS-CoV-2 peptides in the blood. The assay setup consisted of four blood collection tubes, two of which, SARS-CoV-2 Antigen 1 (Ag1) and SARS-CoV-2 Antigen 2 (Ag2), were coated with a combination of SARS-CoV-2 Spike-derived specific antigens that stimulate lymphocytes in the whole blood. Antigen 1 stimulates CD4+ cells, whereas Ag2 stimulates both CD4+ and CD8+ cells. The other two tubes (Nil and Mitogen) were used as negative and positive controls, respectively. Heparinized blood specimens were transferred to Quantiferon blood collection tubes and incubated at 37 °C for 16 to 24 h. After incubation, samples were centrifuged at 3000 rpm for 15 min to separate the plasma. IFN-γ was measured in these plasma samples using an enzyme-linked immunosorbent assay (Quantiferon ELISA, Qiagen). The IFN-γ values of the negative controls were subtracted from those of the stimulated samples. A sample was considered positive when the IFN-γ value was greater than 0.15 IU/mL. Before the administration of the third dose, T cell response was assessed in 44 participants. After the booster dose T-cell response was assessed in 50 recipients (including 40 of the former 44 tested). Specifically, in 40 individuals, cellular immunity was measured before and after the third dose.

### 2.5. Statistical Analysis

Median values, 25th, and 75th percentiles were used to describe anti-RBD levels. All samples below LoD 6.8 AU/mL were assigned the value 6.8 AU/mL. A Poisson regression model with robust sandwich standard error was used to identify factors associated with immune response after the third dose. All statistical analyses were performed using Stata 17.

## 3. Results

The demographic and clinical characteristics of the 144 individuals are presented in Table 1. The majority (65.3%) was male, and the mean (SD) age was 55.8 (13.8) years. The median (25th, 75th) time between the second dose and transplantation was 9.3 (3.1, 16.1) years. Only 5 (3.5%) participants were transplanted less than a year before vaccination, 64 (44.4%) were transplanted 1–9 years before, and 75 (52.1%) had been transplanted more than 9 years before vaccination.

A total of 136/144 (94.4%) were kidney transplant recipients, 7 (4.9%) were heart transplant recipients, and 1 patient was a kidney and heart recipient. The patients, in general, had well-preserved kidney function with a mean (SD) creatinine level of 1.5 (0.6) mg/dL and a mean (SD) eGFR of 54 (18.7) mL/min. Most participants received an immunosuppressive regimen with a calcineurin inhibitor, mostly tacrolimus (75.7%) and, less frequently cyclosporine A (24.3%), in combination with an antimetabolite, mycophenolate acid, in 71.5% and azathioprine in 2.8% of patients. Only 14 (9.7%) of patients received the mammalian target of Rapamycin (mTOR) inhibitor everolimus, while the mean (SD) trough levels of tacrolimus and everolimus were 6.0 (1.1) and 5.7 (1.5) ng/mL, respectively. Most of the participants also received corticosteroids in low maintenance dose (71.5%). Overall, SOT recipients had been transplanted for a long time, received maintenance immunosuppression with two or three immunosuppressive drugs, and retained an adequate renal function.

About two-thirds of patients (65.3%) received two doses of BNT162b2 and the remaining (34.7%) patients received the mRNA1273 vaccine. All patients received a third dose with the mRNA1273 vaccine, due to the vaccination policy based on our previous study, which demonstrated higher immunogenicity among individuals vaccinated with two doses of mRNA1273 compared to those with the BNT162b2 vaccine, as well as higher median antibody levels [12].

### 3.1. Patients with Natural Immunity

We measured the anti-nucleocapsid, anti-(N) IgG antibodies after the third dose, and ten patients (10/144, 6.9%) tested positive. Notably, all of them had a positive history of previous COVID-19, as reported in the questionnaire, and were subsequently excluded from further analysis.

### 3.2. Humoral Response at Three Different Time Points

**Figure 1:**
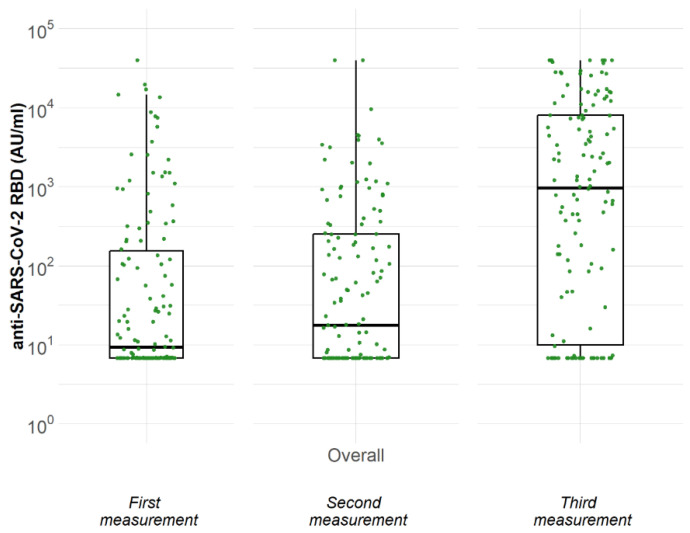
Concentration of anti-SARS-CoV-2 RBD IgG antibodies at three time points after the first dose of vaccine (excluding those with positive anti-N).

As presented in Table 2 and in Figure 1, only 33.6% (45 out of 134) of recipients who received the initial vaccination with two doses tested positive for anti-SARS-CoV-2 RBD-IgG antibodies, with a median (25th, 75th) antibody titer of 9 (7, 161) AU/mL. Approximately 3 months after the second dose, 41.8% (56 out of 134) tested positive with a median (25th, 75th) antibody titer of 18 (7, 251) AU/mL. Remarkably, when tested after administration of the third dose, a positive antibody response was detected in 69.4% (93 out of 134) recipients, with a median (25th, 75th) titer of 966 (10, 8027) AU/mL.

As presented in Table 3, among the covariates assessed, antimetabolite-free and steroid-free immunosuppression were the factors independently associated with anti-N positivity. On the other hand, our analysis did not find age, age at transplantation, time from transplantation, gender, and vaccine type of the primary vaccination to be independently associated with the immune response after the third dose. From univariable analysis, heart transplantation, in comparison to kidney transplantation, was associated with anti-N positivity.

### 3.3. T-Cell Response before and after Booster Vaccination

Before the administration of the third dose, only 5 (11.4%) of the 44 randomly selected participants had a positive response. Following the administration of the third dose, T-cell response was assessed in 50 recipients (including 40 of the former 44 tested) with 21 (42%) of them testing positive.

Specifically, in 40 individuals, cellular immunity was measured before and after the third dose. After the administration of the second dose, 87.5% (35/40) had a negative specific T-cell response. Following administration of the third dose, 34% (12/35) of those who had previously had a negative response displayed a positive response, while 66% (23/35) remained negative even after the third dose (Table 4).

Finally, as shown in Table 5, 81% (17/21) of the recipients with a positive T-cell response after the third dose also had a positive antibody response, while 19% (4/21) had a positive T-cell response despite a negative humoral response.

### 3.4. Safety and Tolerability of the Third-Booster Dose

Among the vaccine recipients, 82% reported at least one adverse event after the third dose. We observed events of mild severity, without allergic reactions or hospitalization for local or systemic adverse events.

Pain at the injection site was the most frequent adverse effect, reported by 91 (73.4%) recipients. The second most frequent adverse effect was fatigue in 24 (19.4%) recipients, followed by fever in 22 (18.0%), myalgia in 9 (7.3%), and headache in 6 (4.8%) recipients.

## 4. Discussion

Herein, we demonstrate a delayed humoral response after the second dose of SARS-CoV-2 vaccine, with only 33.6% of recipients testing positive for anti-SARS-CoV-2 RBD-IgG antibodies one month after administration of the second dose. Interestingly, at three months after the second dose, 41.8% of participants tested positive. There are inconclusive data regarding this apparently delayed humoral response after two doses of mRNA vaccines in SOT recipients. A study by Alejo J et al. included 312 SOT recipients and demonstrated relatively stable antibody titer over time, with seropositivity rates of 63% at 1 month after the second dose of mRNA vaccine, 72% at 3 months, and 73% at 6 months, while 7% of seropositive patients at 1 month became seronegative at 6 months [17]. These findings suggest that the mechanisms involved in humoral immunity responses are not yet clearly defined, and thus further research is required to clarify the immunogenicity of the mRNA vaccines.

Booster vaccination is a well-established method to improve the rates of serological response in immunosuppressed patients. We found a significant augmentation of the humoral response after the booster dose with seropositivity rates of 69.4%. In a recent meta-analysis, Sakuraba A. et al. analyzed 44 observational studies, including 6158 SOT recipients. After one and two doses of the vaccine, serologic response rates were 8.6% (95% CI 6.8–11.0) and 34.2% (95% CI 30.1–38.7), respectively, while a third dose improved the rate to 65.6% (95% CI 60.4–70.2) [18]. Moreover, recently published results showed that this positive effect of booster vaccination on serological response is cumulative and continues with fourth and even fifth vaccination, resulting in a cumulative response rate of 88.7% after five vaccinations [19]. Although a positive anti-RBD response correlates with neutralization assays, it was reported that SOT recipients with a positive anti-RBD response still had undetectable Omicron-specific neutralizing antibody after the third booster dose of a mRNA vaccine. This fact underlines the importance of cellular immunity against newer variants of concern in frail and immunosuppressed populations [20].

In our study, we assessed specific T-cell response in a subset of randomly selected recipients, evaluating IFN-γ secretion by CD4+ and CD8+ lymphocytes in response to SARS-CoV-2 peptides, before and after the administration of the third dose. Before the administration of the third dose, only 11.4% (5/44) of them had a positive response, while, after the third dose, 42% (21/50) of them tested positive. There are limited data regarding T-cell response after a third dose of mRNA vaccines. This is attributed, in part, to the complexity and availability of the techniques used for the measurement of virus-specific T-cell immune responses compared to anti-spike IgG antibody levels. Furthermore, different assays have been utilized to assess T-cell response and their results do not necessarily correlate, hindering comparisons between different studies [21]. To the best of our knowledge, our study is one of the largest assessing cellular immunity after the third dose of vaccination for COVID-19 in SOT recipients. The reported augmentation of the T-cell response after the third dose in our study agrees with other studies. Stumpf J et al., in a single-center study, investigated the humoral and cellular response after 3 doses of BNT162b2 vaccine in 48 of the 78 KTRs participants. The reported cellular response was present in 26% (9/35 patients, no results were reported for 13 patients) and 40% showed a total humoral response [10].

In our subgroup of 40 individuals with measured T-cell response before and after the third dose, 87.5% (35/40) had a negative specific T-cell response, before the third dose. Following administration of the third dose, from those who previously had a negative response, 34% (12/35) displayed a positive response. Another interesting finding of our study was that 19% (4/21) of the SOT recipients with a positive cellular response after the third dose had a negative humoral response; that which initially seems contradictory, is reported by other investigators, too [22].

There has been an ongoing interest in research about the complex interrelationship between B-cell and T-cell immunity, especially due to the emergency of different variants of concern including Delta (B.1.617.2) and Omicron (B.1.1.529) [23]. Studies in immunocompetent patients showed that a robust T-cell response has been associated with a less severe disease course in COVID-19 patients [24]. Furthermore, it is known that T cells, specifically the follicular helper CD4 T-Cells (Tfh), participate in effective B-cell maturation and germinal center B-cell differentiation in memory and antibody-secreting cells, which induces a strong and durable antibody response [25]. Taking those parameters into consideration, the assessment of humoral response after vaccination using the measurement of antibodies against the spike protein’s RBD could underestimate the immunogenicity of SARS-CoV-2 vaccines. In particular in the high-risk population of SOT recipients under immunosuppressive treatment, the combined analysis of humoral and cellular immunity could be part of an individualized management strategy [26].

A recently published study from our center reveals that protection against severe disease might be associated with vaccine-induced cellular immunity. The majority of the infected KTRs were vaccinated with at least three doses of the available mRNA vaccines and had low levels of anti-SARS-CoV-2 antibody titers before the time of infection. Despite that, the percentage of severe disease was low, and one could hypothesize that protection against severe disease could be partially associated to vaccine-induced cellular immunity [24,27].

Finally, our study showed that antimetabolite use in the immunosuppressive regimen is associated with impaired immunogenicity, something which is in agreement with data from other studies. In a recent meta-analysis by Meshram et al., including 112 studies with a total of 15,391 SOT recipients and 2844 healthy controls, mycophenolic acid (MPA) was associated with higher risk for humoral nonresponse (log OR = 1.42 [1.21–1.63], I^2^ = 63.06%) [7]. Pausing MPA at the time of vaccination has also enhanced serological response in no responders KTRs [19]. Despite the reported association of impaired immunogenicity and use of MPA, these data should be addressed carefully, taking into consideration the possible detrimental complications of MPA discontinuation. A recent joint statement of the American Society of Transplant Surgeons, the American Society of Transplantation, and the International Society for Hearn and Lung Transplantation, asserts that, despite data that corelate antiproliferative agents with impaired antibody response after vaccination, there is no reliable guide for the adjustment of immunosuppression in anticipation of vaccine responses [28]. As far as other factors are concerned, male gender, younger age, and later period of transplantation, were not correlated with anti-N positivity after booster dose. In the aforementioned Meshram et al. metanalysis, with thousands of SOT recipients accessed after their third dose, increasing age was a factor for decreased humoral response and also earlier period of transplantation showed a trend toward lower response. In addition, SOT recipients with lower eGFR had higher chances of nonresponse compared to those with a better eGFR, and the use of calcineurin inhibitor (CNI) (but not CNI trough levels) was associated with higher risk of nonresponse [7].

We consider our study’s strong points to be the relatively large number of SOT recipients in our cohort with three consecutive measurements in a strict protocol, providing useful data on humoral response during the study period, after primary and booster vaccination with mRNA vaccines. Another strong point of our study is the significant number of recipients with measured specific T-cellular response, which plays an important role in the immune response against SARS-CoV-2 infection and variant breakthrough infections. Its limitations include the absence of a healthy control group, the lack of information about immunogenicity after the next booster doses, and the absence of measuring neutralizing antibody titers against newer variants of concern such as Omicron. The characteristics of the patient cohort, such as the dominance of male subjects (65%), the dominance of Pfizer-BioNTech vaccine as primary vaccination (65%), and the dominance of kidney transplant recipients (94%), could also affect the distribution of observed data and the interpretation of the findings.

## 5. Conclusions

To conclude, herein we report an augmentation of humoral response three months after the second dose, and an even stronger humoral and cellular response after a third booster dose, of the mRNA vaccine in a cohort of 144 SOT recipients. Our results may contribute to a deeper comprehension of mRNA-vaccines-induced immune responses, both humoral and cellular, in this high-risk population and underline the effects of booster vaccination as COVID-19 takes on endemic characteristics. Further research is necessary to determine the optimal vaccination strategy for high-risk populations, considering that COVID-19 will be present in the next years and booster doses are a possible way to preserve high rates of immunity against severe disease.

## Figures and Tables

**Table 1:** Demographic, clinical, and immunogenic profiles of a cohort of solid organ transplant recipients who received booster (3rd) vaccinations.

	N = 144
Gender, n (%)	
Male	94 (65.3)
Female	50 (34.7)
Age (years) mean (S.D.) ^1^	55.8 (13.8)
Age (years) median (25th, 75th) ^2^	56.5 (45.5, 68.7)
Age at transplantation (years) mean (S.D.) ^1^	45.1 (13.2)
Age at transplantation (years) median (25th, 75th) ^2^	46.5 (35.7, 55.0)
Time period between transplantation and second dose (years), median (25th, 75th) ^2^	9.3 (3.1, 16.1)
Time period between transplantation and second dose (years), n (%)	
<1	5 (3.5)
1–9	64 (44.4)
>9	75 (52.1)
Organ transplant, n (%)	
Kidney	136 (94.4)
Heart	7 (4.9)
Kidney/Heart	1 (0.7)
**Kidney function**	
Creatine (mg/dL) mean (S.D.) ^1^	1.5 (0.6)
Creatinine (mg/dL) Median (25th, 75th) ^2^	1.4 (1.1, 1.7)
eGFR (mL/min) mean (S.D.) ^1^	54.0 (18.7)
eGFR (mL/min) median (25th, 75th) ^2^	53 (42, 65)
**Immunosuppression**	
Tacrolimus, n (%)	
Yes	109 (75.7)
No	35 (24.3)
Tacrolimus levels (ng/mL) mean (S.D.) ^1^	6.0 (1.1)
Tacrolimus levels (ng/mL) median (25th, 75th) ^2^	6.0 (5.4, 6.7)
Mycophanolate acid, n (%)	
Yes	125 (86.8)
No	19 (13.2)
Azathioprine, n (%)	
Yes	4 (2.8)
No	140 (97.2)
Corticosteroids, n (%)	
Yes	103 (71.5)
No	41 (28.5)
Cyclosporine A, n (%)	
Yes	35 (24.3)
No	109 (75.7)
Everolimus, n (%)	
Yes	14 (9.7)
No	130 (90.3)
Everolimus levels (ng/mL) mean (S.D.) ^1^	5.7 (1.5)
Everolimus levels (ng/mL) median (25th, 75th) ^2^	5.9 (4.5, 6.6)
Vaccine Type for the first two doses, n (%)	
Pfizer–BioNTech	94 (65.3)
Moderna	50 (34.7)

^1^ Standard deviation, ^2^ 25th–75th percentile. Abbreviations: eGFR, estimated glomerular filtration rate.

**Table 2:** Concentration of anti-SARS-CoV-2 RBD IgG antibodies at three time points after the first dose of vaccine (excluding those with positive anti-N).

	Measurement N = 134		
	First	Second	Third
Antibodies (AU/mL), median (25th, 75th) ^1^	9 (7, 161)	18 (7, 251)	966 (10, 8027)
Antibody levels, n (%)			
Negative (<50 AU/mL)	89 (66.4)	78 (58.2)	41 (30.6)
Positive (≥50 AU/mL)	45 (33.6)	56 (41.8)	93 (69.4)

^1^ Interquartile range (25th–75th percentile).

**Table 3:** Factors associated with the incidence of positive anti-N among solid organ transplant recipients, excluding those with positive anti-N, N = 134.

Variable	Univariable RR (95% CI)	*p*-Value	Adjusted Multivariable RR (95% CI)	*p*-Value
Age (years)	1.00 (0.99–1.01)	0.614		
Age at transplantation (years)	1.00 (1.00–1.01)	0.283	1.01 (1.00–1.01)	0.236
Time from transplantation up to 2nd dose of vaccination (years)	0.99 (0.98–1.01)	0.382		
Gender		0.145		
Male	Ref.		Ref.	
Female	0.83 (0.64–1.07)		0.80	0.066
Vaccine		0.204		
Pfizer/Biontech	Ref.			
Moderna	1.15 (0.92–1.44)			
CNI = (Tacrolimus + Cyclosporine)		0.698		
Yes	Ref.			
No	0.92 (0.61–1.40)			
Mycophenolic Acid		<0.001		<0.001
Yes	Ref.		Ref.	
No	1.55 (1.35–1.77)		1.48 (1.24–1.77)	
Corticosteroids		0.001		0.048
Yes	Ref.		Ref.	
No	1.40 (1.15–1.71)		1.24 (1.00–1.53)	
Everolimus		0.001		
Yes	Ref.			
No	0.72 (0.59–0.87)			
Organ transplanted		<0.001		
Kidney	Ref.			
Heart	1.48 (1.31–1.67)			

Abbreviations: RR, risk ratio; CI, confidence interval; CNI, calcineurin inhibitors.

**Table 4:** Cellular immunity based on third dose of vaccine, excluding those with positive anti-N, N = 40.

Cell Immunity	After 3rd Dose		
Before 3rd Dose	No	Yes	Total
No	23 (100.0%)	12 (70.6%)	35 (87.5%)
Yes	0 (0.0%)	5 (29.4%)	5 (12.5%)
Total	23 (100.0%)	17 (100.0%)	40 (100.0%)

**Table 5:** Cellular immunity among solid organ transplant recipients in Greece based on antibodies levels after third dose of vaccine, excluding those with positive anti-N, N = 50.

	Cell Immunity		
Antibody levels after 3rd dose	No	Yes	Total
Negative (<50 AU/mL)	11 (37.9%)	4 (19.0%)	15 (30.0%)
Positive (≥50 AU/mL)	18 (62.1%)	17 (81.0%)	35 (70.0%)
Total	29 (100.0%)	21 (100.0%)	50 (100.0%)

## Data Availability

The data presented in this study are available on request from the corresponding author.
